# ADVERSE CHILDHOOD EXPERIENCES AND ALZHEIMER’S DISEASE/DEMENTIA RISK FACTORS AMONG TRANSGENDER ADULTS

**DOI:** 10.1093/geroni/igae098.2484

**Published:** 2024-12-31

**Authors:** Ethan Cicero, Jobina Chiow, Jason Flatt

**Affiliations:** Emory University, Quincy, Massachusetts, United States; Emory University, Atlanta, Georgia, United States; University of Nevada Las Vegas, Las Vegas, Nevada, United States

## Abstract

Adverse childhood experiences(ACEs) are associated with an increased risk of developing Alzheimer’s disease and related dementias(ADRD). Compared to the general population, transgender, nonbinary, and other gender expansive people(TNGE) report a higher prevalence of ACEs and ADRD. The relationship between ACEs and ADRD risk factors among TNGE adults has not been explored. 2019-2021 Behavioral Risk Factor Surveillance System(BRFSS) data, representing 16 U.S. states that assessed ACEs, subjective cognitive decline(SCD), and gender identity were used to determine the association between ACEs and ADRD risk factors(coronary heart disease[CHD], depression, diabetes, hearing loss, high cholesterol, hypertension, obesity, SCD, smoking, stroke) among TNGE adults aged 45+(N=206). Chi-squared and adjusted odds ratios(aOR) along with 95% confidence intervals(CI) were calculated, adjusting for BRFSS year, age, race, education, and employment. Obesity(70%), hypertension(56%), high cholesterol(47%) and depression(29%) were the most prevalent risk factors among the sample. Experiencing any ACE was associated with depression and SCD; >4 ACEs was associated with CHD, depression, SCD, and stroke. As the number of ACEs increased, the odds of CHD, depression, SCD, and stroke increased by 29–41%. The odds of depression and SCD were higher with childhood abuse (aOR=3.1, 95%CI:1.7-5.9, p< 0.01; aOR=3.8, 95%CI:1.7-8.3, p< 0.01) or household dysfunction (aOR=5.1, 95%CI:2.4-10.6, p< 0.0001; aOR=4.7, 95%CI:2.0-11.1, p< 0.01). Compared with no ACEs, experiencing 2,3, and >4ACEs were associated with 1.3–5.6x higher odds of depression and SCD. A lifecourse perspective is needed when examining the brain health of TNGE adults. ACE screening/interventions for TNGE youth are needed to help reduce the risk of ADRD later in life.

